# Determinants of Maternal Self‐Medication Practice for Children Under 5 Years of Age: A Community‐Based Study in Dessie, Ethiopia

**DOI:** 10.1155/ijpe/5464338

**Published:** 2026-09-24

**Authors:** Debrnesh Goshiye, Sisay Gedamu, Mitaw Girma, Mulusew Zeleke, Birhanu Goshiye

**Affiliations:** ^1^ Department of Pediatric and Child Health Nursing, College of Medicine and Health Sciences, Wollo University, Dessie, Ethiopia, wu.edu.et; ^2^ Department of Comprehensive Nursing, College of Medicine and Health Sciences, Wollo University, Dessie, Ethiopia, wu.edu.et; ^3^ Department of Adult Health Nursing, College of Medicine and Health Sciences, Wollo University, Dessie, Ethiopia, wu.edu.et; ^4^ Woldia Comprehensive Specialized Hospital, Woldia, Amhara, Ethiopia

**Keywords:** Ethiopia, mothers, self-medication, under-five children

## Abstract

**Background:**

Self‐medication and the irrational use of drugs are major public health concerns worldwide. The concept of self‐medication involves the use of medicines for curative purposes without professional advice. This practice is more disastrous in children, where dosages vary with weight, age, or body surface area. This study is aimed at assessing the determinants of maternal self‐medication practice in children under 5 years of age.

**Methods:**

Community‐based cross‐sectional study was conducted at Dessie Town. A semistructured questionnaire was used to interview mothers of under‐five children. Data entry was done using Epi Data 3.1 and analyzed using SPSS Version 20. Logistic regression was done to identify association between the dependent and independent variables.

**Result:**

The overall prevalence of maternal self‐medication practice for under‐five children was 78.0% (95% CI: 74.7%–81.3%). In the multivariable analysis, mothers with children aged older than 1 year (AOR = 2.74, 95% CI: 1.31–5.72), mothers aged > 35 years (AOR = 8.78, 95% CI: 3.28–23.54), mothers with primary education (AOR = 2.94, 95% CI: 1.07–8.05), student mothers (AOR = 6.52, 95% CI: 1.68–25.27), and mothers with poor knowledge (AOR = 1.83, 95% CI: 1.11–3.01) had significantly higher odds of practicing self‐medication. Parity exhibited bidirectional associations: Having two children increased the odds of self‐medication (AOR = 2.20, 95% CI: 1.32–3.65), whereas having three or more children decreased the odds (AOR = 0.34, 95% CI: 0.16–0.74) relative to mothers with one child.

**Conclusion:**

Maternal self‐medication practice was high.. Hence, measures should be taken to improve mothers′ knowledge, such as health education and awareness creation campaigns for mothers on the risks and potential consequences of self‐medicating under‐five children.

## 1. Background

Self‐medication is an important global health concern, defined as the selection and use of nonprescription medicines by individuals on their own initiative to treat self‐recognized illnesses or symptoms [1–3]. The prevalence of self‐medication is alarmingly high worldwide, ranging from 8.5% to 98% in both developing and developed countries [4, 5]. It is also high in Africa, especially in sub‐Saharan Africa, with a prevalence ranging from 11.9% to 75.7% [6, 7]. In low‐ and middle‐income countries (LMIC), it is estimated that approximately 80% of antibiotics are used outside official healthcare facilities, of which approximately 20%–50% are used inappropriately [8]. A study conducted in Madagascar found the prevalence of self‐medication practices for children by mothers to be 40.99% [9].

Self‐medication practices are of great concern in the case of children, as they are considered to be more vulnerable to the use of medicines. Infancy and early childhood are characterized by rapid growth and development, which differ in terms of many metabolic and physiological actions from adulthood [10, 11]. Children constitute a large proportion of the population in developing countries and are more susceptible to different diseases, specifically diarrhea, vomiting, cough, and fever [12]. Despite various reports of irregular drug use among most communities in developing countries, self‐medication practices in children (particularly those under 5 years of age) have not been well studied [6, 7].

Self‐medication is associated with potential risks for the individual consumer, such as incorrect self‐diagnosis, failure to seek appropriate medical advice promptly, incorrect choice of therapy, failure to recognize special pharmacological risks, inadequate or excessive dosage, excessively prolonged use, incorrect route of administration, risk of dependence and abuse, interactions, warnings, failure to recognize or report adverse drug reactions, excessive dosage, and excessively prolonged use. This is because the user usually has no specialized knowledge of the principles of pharmacology, therapy, or of the specific characteristics of the medicinal product used. At the community level, hazards from self‐medication include improper self‐medication, which is related to an increase in drug‐induced disease, tolerance, resistance in the body, and wasteful public expenditure [13, 14].

The major reasons identified for self‐medication in Africa are illiteracy, minor illness, lack of money, lack of healthcare providers, distance to a healthcare facility, and the often negative attitude of healthcare providers [15]. A study conducted in Uganda showed that one‐third of the caretakers of children under 5 years of age practiced self‐medication for symptomatic children [7]. In another study, the prevalence of self‐medication among children was 56.6% in Brazil and 96% in the Congo [16].

The use of available medications without a doctor′s prescription is linked to inappropriate use and serious adverse drug effects, which are associated with poor health consequences for children [17]. Although self‐medication is common in pediatrics, little is known about self‐medication use in children in Ethiopia. To date, no study has been conducted on self‐medication practices among mothers of children under 5 years of age in Ethiopia. Therefore, this study is aimed at assessing maternal self‐medication practices and the determinant factors for under‐five children in Dessie Town.

## 2. Methods

### 2.1. Study Design, Area, and Period

A community‐based cross‐sectional study was conducted in Dessie city from January 1 to 15, 2023.

### 2.2. Population

All mothers with children under 5 years of age and permanently living in Dessie Town were the source population, and those mothers with children under 5 years of age who had permanently lived in Dessie Town for at least 6 months and were found at the time of data collection were considered to be the study population.

### 2.3. Eligibility Criteria

Mothers of children under the age of five who have lived permanently in Dessie Town for at least 6 months and volunteered to participate were included in the study. Mothers with communication problems, severe illness, mental challenges, or hearing problems were excluded from the study.

### 2.4. Sample Size Determination

The sample size was calculated using a single population proportion formula. Because no previous study has been conducted on maternal self‐medication for children under 5 years of age in Ethiopia, 50% was taken, at a *Z* value of 1.96 and a marginal error of 5%. The sample size was 384, which was multiplied by 1.5 for the design effect and 10% for the nonresponse rate; thus, the final sample size was 633.

### 2.5. Sampling Technique

Multistage cluster sampling was used. Dessie Town was first stratified into five subcities, from which three were selected using simple random sampling (lottery method). Next, one kebele was randomly selected from each chosen subcity using the same lottery approach. The required sample size was allocated proportionally across the three kebeles based on household population data obtained from local health posts. In each kebele, sampling began at a central landmark using a bottle spin to pick a random direction. The first household was selected via a random number [1–10], and subsequent adjacent households were enrolled systematically until reaching the target sample size. To prevent selection bias, collectors enrolled one mother–child pair per household, made up to two same‐day revisits for absent households, and conducted daily supervisory spot‐checks.

### 2.6. Data Collection Procedure

A semistructured pretested questionnaire [18] was used to collect data from mothers via face‐to‐face interviews. All data collectors and supervisors were trained on their responsibilities for describing the purpose of the study, how to collect the data, and telling clients the importance of honest and genuine replies toward the questions. The principal investigator and supervisors strictly followed the overall activities of data collection daily to ensure the completeness of the questionnaire and provide further clarification.

### 2.7. Study Variable

#### 2.7.1. Dependent Variable

• Maternal self‐medication practice

#### 2.7.2. Independent Variables

• Sociodemographic factors (age, religion, marital status, educational status, and occupation).
• Healthcare facility related factors (type of health care facility near to home, waiting time, and traveling time to nearest care facility)
• Knowledge about self‐medication
• Other factors (peer pressure, reason for use, and frequency).

### 2.8. Operational Definitions

Maternal self‐medication means “the selection and use of medicines by their mothers for the treatment of self‐diagnosed ailments of these children; it includes home remedies, drugs given on the advice of a member of a social network (friend, neighbor, grandmothers, etc.), drugs given by any pharmacist/compounder, leftover drugs, or taking them to spiritual healers, hakeem, or homeopath.”

• Practice on self‐medication: The prevalence of self‐medication practice was assessed using a yes/no question or measured by an affirmative response (yes) to the question, “Did you give your under‐five children medicine without the doctor′s permission or advice in the prior 3 months?” [19].
• Knowledge about self‐medication: Maternal knowledge was evaluated using eight binary items (0 = incorrect, 1 = correct; score range: 0–8). Because the knowledge scores were heavily right‐skewed (sample mean = 7.3) and measured in whole integers, a mean‐based cutoff of older than7.3 meant that only participants achieving a perfect score of 8/8 were classified as having “good knowledge,” whereas those scoring ≤ 7 were categorized as having “poor knowledge.” This restrictive, sample‐derived classification was utilized to differentiate fully knowledgeable mothers from those with any knowledge deficits, rather than applying an externally validated clinical threshold.

### 2.9. Data Quality Assurance

The questionnaire was adapted from previously published literature on pediatric self‐medication in similar LMIC contexts. The tool was translated into Amharic (the local language) by fluent bilingual experts and back‐translated into English by an independent translator to ensure consistency. Reliability testing for the eight knowledge items yielded a Cronbach′s alpha of 0.78, indicating acceptable internal consistency. We will provide these Amharic and English versions of the questionnaire when requested.

Training was provided to both the data collectors and supervisors. Data were collected through in‐person interviews. Prior to the data collection, a pretest was conducted in a separate area in Kombolcha Town with 32 participants. The data collectors and supervisors were trained on the data collection instruments and procedures. Strict supervision was conducted during the data‐collection period. The completeness and clarity of the data were checked daily by supervisors and principal investigators, and honest and genuine replies were provided to the questions. The principal investigator and supervisors strictly followed the overall activities of data collection daily to ensure the completeness of the questionnaire and provide further clarification.

### 2.10. Data Analysis Procedure

Data were entered into Epi Data Version 3.1 and exported and analyzed using SPSS Software Version 20. Binary and multiple logistic regression analyses were performed to assess the associations between independent and dependent variables. Independent variables with a *p* value < 0.25 in the bivariable logistic regression were selected for inclusion in the multivariable logistic regression model. Model fitness was evaluated using the Hosmer–Lemeshow goodness‐of‐fit test (*p* > 0.05 indicated a good model fit). Potential confounders were controlled for by including them in the adjusted multivariable model.

### 2.11. Ethical Consideration

Ethical clearance was obtained from the Research and Ethical Review Committee of the College of Medicine and Health Science, Wollo University, with the Reference Number CMHS‐604/12/15. An official letter of permission from the college was submitted to the healthcare facilities in Dessie Town and to the concerned bodies to conduct the research. A written informed consent was obtained from each mother. All the collected data was kept confidential, and no one except the members of the research team had access to the collected information.

## 3. Result

### 3.1. Description of Study Participants

The total number of participants in the study was 623 pairs of mothers and children under 5 years of age (98.4% participation rate). Among them, half (50.4%) of the study participants had a male child, and approximately 80.4% of the children were older than 1 year. About 62.1% of mothers were aged 25–35 years, 82.7% were married, and 34.8% had an educational level of college and above. Most of the participating mothers (64.5%) were housewives in their occupation, and nearly half (50.6%) of the mothers had one child. Regarding their fathers, approximately half (49.6%) were government employees, as shown in Table 1).

**Table 1:** Sociodemographic characteristics of the study participant in Dessie Town, Ethiopia, 2023 (*N* = 623).

Variable	Frequency (*N*)	Percent (%)
Child sex		
Female	309	49.6
Male	314	50.4
Child age		
< 1 year	122	19.6
Older than 1 year	501	80.4
Mother age		
< 25 years	143	23.0
25–35 years	387	62.1
> 35 years	93	14.9
Educational status of mother		
No formal education	42	6.7
Grades 1–8	152	24.4
Grades 9–12	212	34.0
College and above	217	34.8
Marital status		
Single	15	2.4
Married	515	82.7
Widowed	43	6.9
Divorced	50	8.0
Occupation of mother		
Housewife	402	64.5
Student	14	2.2
Government employee	158	25.4
Other	49	7.9
Occupation of father		
Government employed	309	49.6
Merchant	285	45.7
Daily laborer	29	4.7
Number of children		
One child	315	50.6
Two child	167	26.8
Three and more	141	22.6

*Note:* Other = merchant and daily laborer.

### 3.2. Healthcare Facility Access–Related Factors

As stated by the participants, half of them had access to health centers. More than 4/5th of the participants stated that the time taken to reach the nearest healthcare facility was less than 30 min, and around half of the participants stated that the waiting time at the healthcare facility was less than 15 min, as shown in Table 2.

**Table 2:** Healthcare facility access–related factors.

Variable	Frequency (*N*)	Percent (%)
Nearest healthcare facility		
Hospital	55	8.8
Health center	312	50.1
Health post	108	17.3
Private clinic	148	23.8
Time taken to reach the nearest health care facility		
Less than 30 min	509	81.7
Greater than 30 min	114	18.3
Waiting time at health care facility		
< 15 min	299	48
15–30 min	269	43.2
> 30 min	55	8.8

*Note:* Reason to self‐medicate the child (*N* = 486).

Among the 623 study participants, 78% described that they had self‐medicated their under‐five child. About majority (85.4%) of the participants stated family/peer pressure as the main reason for self‐medicating their under‐five child, followed by having an emergency illness (40.7%), as shown in Table 3.

**Table 3:** Reason for self‐medicating the child (*N* = 486).

Reason	Frequency	Percent (%)
No time to go health facility	68	14
Illness was simple	146	30
Heath facility was far	188	38.7
Illness was emergency	198	40.7
Medication was cheap	161	33.1
Know the medication well	102	21
Family/peer pressure	415	85.4

*Note:* Percentages do not sum to 100% because multiple responses were permitted.

### 3.3. Signs/Symptoms Treated Through Self‐Medication

**Figure 1:**
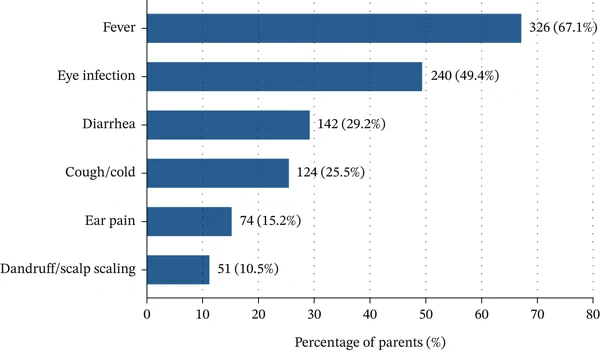
Signs/symptoms of the child that are treated through self‐medication by their mothers (*N* = 486). Note: Percentages do not sum to 100% because multiple responses were permitted.

Among the participants who self‐medicated their children under 5 years of age, the most commonly treated sign/symptom was fever (67.1%), followed by eye infection (49.4%) and diarrhea (29.2%), as shown in Figure 1.

### 3.4. Knowledge About Self‐Medication

**Figure 2:**
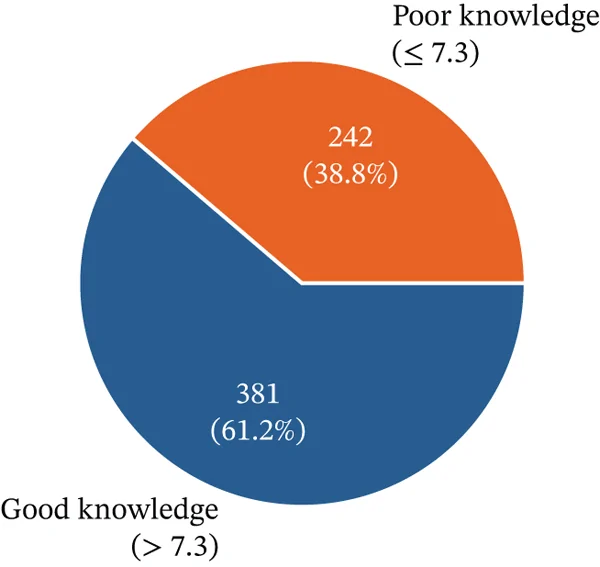
Mothers level of knowledge about self‐medication (*N* = 623). Factors associated with maternal self‐medication practice.

Based on the questions used to assess the knowledge level of the study participants, a mean value of 7.3 was calculated. Out of 623 participants, 242 (38.8%) of them scored less than or equal to 7.3, indicating poor knowledge regarding self‐medication among these mothers, as shown in Figure 2.

Based on the multivariate analysis, mothers with children aged older than1 year, mothers aged 35 years, mothers with education of Grades 1–8, mothers who are students by occupation, those who have two or three or more children, and those with poor knowledge of self‐medication were found to have a significant association with self‐medication of their children under 5 years of age.

Mothers with a child aged greater than 1 year were more likely to self‐medicate their under‐five than those with a child less than 1 year old. Mothers aged greater than 35 years were more likely to self‐medicate their children than those aged of less than 25 years.

Mothers with an education status of Grades 1–8 were more likely to self‐medicate their child compared with those who have no formal education. Compared with housewife mothers, mothers with student occupations were more likely to self‐medicate their child. In contrast, mothers with the occupations of others (daily laborer and merchant) had significantly lower odds of self‐medicating their children compared with housewife mothers.

In addition, maternal parity displayed a nonlinear association with maternal self‐medication practice. Mothers with two children had more than two‐fold higher odds of self‐medicating their child compared with those with one child (AOR = 2.196, 95% CI: 1.320–3.654, *p* = 0.002). On the contrary, mothers with three or more children had 65.7% lower odds of practicing self‐medication compared with mothers with a single child (AOR = 0.343, 95% CI: 0.159–0.742, *p* = 0.007) as shown in Table 4.

**Table 4:** Association of independent variables with maternal self‐medication practice for their under‐five children in Dessie Town, Ethiopia, 2023.

Variables	Maternal self‐medication practice		Odds ratio (95% CI)			
	Yes	No	Crude	*p*	Adjusted	*p*
Child age						
< 1 year	108	14	Reference		Reference	
Older than 1 year	378	123	2.51 (1.39, 4.54)	0.002	2.74 (1.31, 5.72)	0.007
Mother age						
< 25 year	122	21	Reference		Reference	
25–35 year	307	80	1.51 (0.90, 2.56)	0.121	1.70 (0.87, 3.34)	0.121
> 35 year	57	36	3.67 (1.97, 6.84)	< 0.001	8.78 (3.28, 23.54)	< 0.001
Mother educational status						
No formal education	35	7	Reference		Reference	
Grades 1–8	101	51	2.53 (1.05, 6.08)	0.039	2.94 (1.07, 8.05)	0.036
Grades 9–12	184	28	0.76 (0.31, 1.88)	0.553	0.73 (0.25, 2.18)	0.578
College and above	166	51	1.54 (0.64, 3.67)	0.334	1.58 (0.56, 4.44)	0.384
Mother occupation						
Housewife	308	94	Reference		Reference	
Student	7	7	3.277 (1.121, 9.579)	0.030	6.516 (1.68, 25.27)	0.007
Government employee	129	29	0.737 (0.463, 1.172)	0.197	0.761 (0.373, 1.55)	0.451
Other	42	7	0.546 (0.237, 1.256)	0.155	0.37 (0.14, 0.982)	0.046
Number of children						
One child	264	51	Reference		Reference	
Two child	109	58	2.754 (1.779, 4.266)	< 0.001	2.196 (1.32, 3.654)	0.002
Three and more child	113	28	1.283 (0.770, 2.138)	0.340	0.343 (0.159, 0.742)	0.007
Knowledge level on self‐medication						
Poor	163	79	2.699 (1.832, 3.977)	< 0.001	1.831 (1.114, 3.010)	0.017
Good	323	58	Reference		Reference	

*Note:* Interpret student subgroup findings cautiously due to the small sample size and wide confidence interval.

## 4. Discussion

Self‐medication is a global phenomenon and potential contributor to human pathogen resistance to antibiotics. The adverse consequences of such practices should always be emphasized to the community and steps to curb it [18]. The study is aimed at determining the proportion of mothers self‐medicating their under‐five children and the factors associated with this practice.

Based on the study finding, the proportion of maternal self‐medication practice to under‐fives was 78%. It is in line with the prevalence of self‐medication in Africa, which ranged from 12.1% to 93.9%, with a median prevalence of 55.7% [6] as well as reports from Lahore, Pakistan, and Rwanda [11, 20]. This shows that self‐medication in children is a universal practice with some differences between countries.

This result was higher than the study conducted in Romania, Tanzania, São Paulo, China, Indonesia, Egypt, Madagascar, Uganda, and Assam [7, 9, 10, 16, 17, 19, 21–25]. This variation may be due to the difference in age of the study population, study setting, and the socioeconomic and cultural attributes of the population studied. On the other hand, our prevalence rate was lower than figures reported from Nigeria, the Democratic Republic of Congo, Saudi Arabia, and Somaliland [12, 26–28]. These discrepancies can be explained by demographic and methodological variations. Several of those comparative studies evaluated broader pediatric age ranges (including older school‐aged children) or were conducted in remote rural areas with severe primary healthcare infrastructure shortages. In line with regional findings, maternal decision‐making was heavily influenced by peer networks and acute symptom presentation. Perceiving an illness as urgent or acute was a major driver, consistent with observations from Western Nigeria. [29]. The primary symptoms for which mothers initiated self‐medication were fever, eye infection, diarrhea, and cough, which mirror those identified in other regional studies [12, 21, 27, 30]. This is consistent with the study in Africa, which showed that febrile illness was one of the most common indications to use self‐medication, even though it does not describe specifically under‐five children [6].

These treated symptoms correspond to the primary clinical presentations of common childhood illnesses highlighted in World Health Organization guidelines [31]. We observed a higher prevalence and pattern of common signs/symptoms treated through self‐medication than reported in Uganda and Somaliland [7, 28]. The difference in symptom patterns and burden may be explained by the difference in study setting. The population in the Nigerian study included school‐going children, whose burden of diseases differs greatly from those less than 5 years old enrolled in our community study.

Our findings indicate that maternal self‐medication practice increased with the age of the child; children older than 1 year had significantly higher odds of receiving self‐medication compared with infants under 1 year. This finding reflects a general trend in the majority of studies reporting higher prevalence of self‐medication use in 3‐ to 5‐year‐old children, like in Uganda [7]. As mentioned above, it may be because caretakers are more cautious about administering drugs to very young children with various fears or reservations.

Conversely, mothers older than 35 years had significantly higher odds of engaging in maternal self‐medication practice compared with those less than 25 years of age. We hypothesize that older mothers may draw upon accumulated caregiving experiences or past clinical visits when initiating self‐medication practice, as reported in similar low‐resource settings [12, 20]. However, as maternal confidence was not directly evaluated in this study, qualitative exploration is needed to further examine these potential behavioral associations. Additionally, mothers with an education status of Grades 1–8 had significantly higher odds of engaging in maternal self‐medication practice compared with those who have no formal education. This finding aligns with research from India [30]. Mothers with basic formal education may have greater exposure to general health messages or drug product names, potentially perceiving over‐the‐counter administration as an accessible option for minor complaints compared with mothers without formal schooling.

Although student status was associated with increased odds of maternal self‐medication practice, this finding must be interpreted with caution because of small sample size and wide confidence interval. But this can be hypothesized that student mothers face strict academic schedule constraints and possess higher digital information exposure, which might incline them toward over‐the‐counter purchases rather than routine clinic visits.

Conversely, mothers working as daily laborers or merchants had lower odds of practicing self‐medication compared with housewives. This association may reflect differences in daily movement patterns or greater proximity to community health workers and health posts during working hours.

Mothers′ parity displayed a nonlinear association with self‐medication. Mothers with two children were more likely to self‐medicate than those with one child, whereas mothers with three or more children were significantly less likely. We speculate that managing two young dependents increases domestic time burdens and childcare strain, making quick purchases at neighborhood drug shops more convenient than taking multiple children to a clinic. This aligns with broader pediatric literature demonstrating that parental stress and behavioral management capacity directly shape caregiver health‐seeking decisions [32]. Conversely, as family size reaches three or more children, cumulative lifetime exposure to clinical consultations enhances caregivers′ awareness of pediatric drug risks, whereas strong professional values among nursing personnel foster trust to encourage formal facility utilization over self‐medication. This contrasts with findings in Rwanda [20]—where higher parity correlated with increased self‐medication—may stem from sample age differences, as the Rwandan study included all pediatric age groups whereas ours focused exclusively on vulnerable under‐fives.

Mothers with poor knowledge regarding self‐medication were significantly more likely to practice it, consistent with evidence from Tanzania and Somaliland [28, 33]. Insufficient knowledge regarding correct pediatric dosages, drug interactions, and side effects may cause caregivers to underestimate the risks of unprescribed pharmaceutical use.

## 5. Conclusion

The overall prevalence of maternal self‐medication for under‐five children was 78%. Maternal self‐medication was significantly higher among mothers with children aged older than1 year, mothers aged older than 35 years, mothers with primary education (Grades 1–8), student mothers, and mothers with poor self‐medication knowledge. Maternal parity showed opposing directions of association: Having two children was associated with higher odds of self‐medication, whereas having three or more children was protective, showing lower odds compared with having one child. Conversely, working as a daily laborer or merchant was associated with decreased odds of self‐medication. We recommend targeted health education campaigns focusing on drug risks, particularly aimed at mothers with basic education, older mothers, and those managing multiple young dependents while strengthening primary healthcare networks by equipping community and pediatric nurses with evidence‐based practice competencies to deliver sound child health education [34].

## 6. Limitation of the Study

This study may have limitations, including dependence on the memory of the mothers regarding medication use and the employment of a cross‐sectional study design, in which causal relationships between variables cannot be established. Also, the analyses were based on self‐reports with the possibility of over‐ and underreporting. Additionally information regarding specific drug categories (e.g., antipyretics like paracetamol, antibiotics, cough syrups, and oral rehydration salts) was not fully captured in our study, so we recommended that future studies include itemized tracking of specific pharmacological classes.

## Author Contributions

D.G. and B.G. participated in the development proposal, method, and objectives of this study, ready the questionnaire, analyzed, and wrote the first draft of the manuscript. S.G., M.G., M.Z., and B.G. managed the development of the study design, carried out a literature review, and helped in manuscript writing. All authors developed and supervised the research, edit, and prepared the manuscript for publication.

## Funding

No funding was received for this manuscript.

## Disclosure

All authors read and approved the manuscript.

## Ethics Statement

Ethical clearance was gained from the Research and Ethical Review Committee of the College of Medicine and Health Science, Wollo University. To each subcity of Dessie Town, an official letter of permission from the college was submitted. After full permission, a written informed consent was taken from each mother. Confidentiality of the collected data was maintained by putting it in a secured place, and no one except the members of the research team had access to the collected information.

## Conflicts of Interest

The authors declare no conflicts of interest.

## Data Availability

The data that support the findings of this study are available on request from the corresponding author. The data are not publicly available due to privacy or ethical restrictions.
